# Supplementary material for: A whole-animal phenotypic drug screen identifies suppressors of atherogenic lipoproteins
Source: eLife. 2026 Aug 27;14:RP105314. doi: 10.7554/eLife.105314 (PMC13521566; doi:10.7554/eLife.105314)
Supplement: Supplementary file 2 [file elife-105314-code1.zip › JHDL_primary_result/Calcipotriene/Calcipotriene_JHDL_32_013_048_2020_01_17_12_09.pdf]

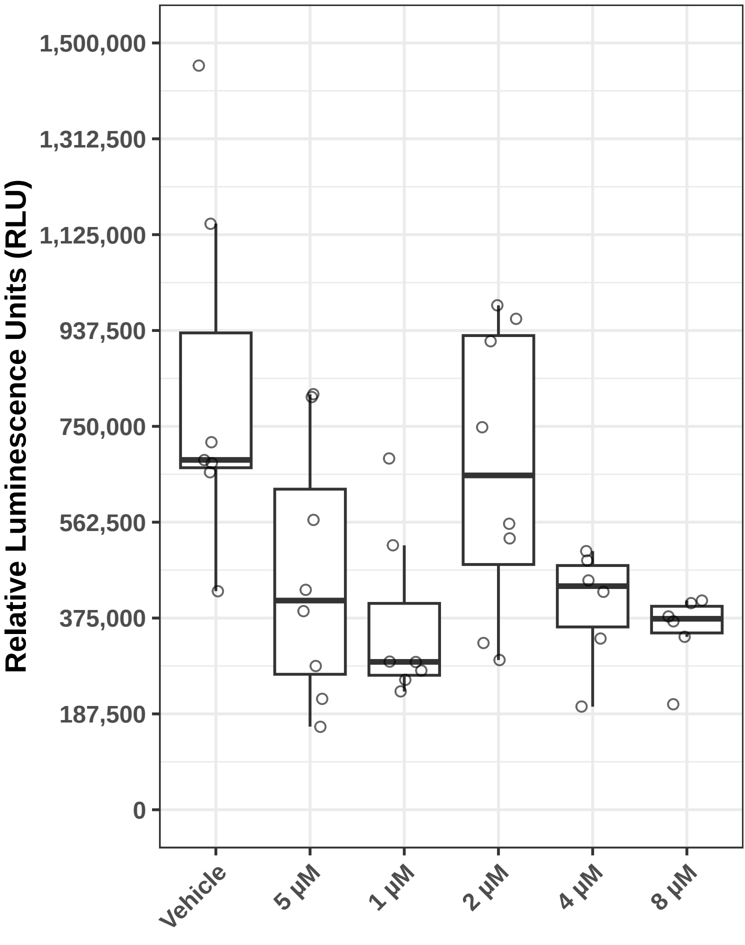

Relative Luminescence Units (RLU)
1,500,000
1,312,500
1,125,000
937,500
750,000
562,500
375,000
187,500
0
Vehicle
5 µM
1 µM
2 µM
4 µM
8 µM
